# Fear Learning Regulates Cortical Sensory Representations by Suppressing Habituation

**DOI:** 10.3389/fncir.2017.00112

**Published:** 2018-01-10

**Authors:** Shea N. Gillet, Hiroyuki K. Kato, Marissa A. Justen, Mandy Lai, Jeffry S. Isaacson

**Affiliations:** ^1^Department of Neurosciences, Center for Neural Circuits and Behavior, University of California, San Diego, La Jolla, CA, United States; ^2^Department of Biological Sciences, University of California, San Diego, La Jolla, CA, United States

**Keywords:** auditory cortex, fear conditioning, interneurons, GCaMP, learning and memory, cortical circuits, somatostatin

## Abstract

Projections from auditory cortex to the amygdala are thought to contribute to the induction of auditory fear learning. In addition, fear conditioning has been found to enhance cortical responses to conditioned tones, suggesting that cortical plasticity contributes to fear learning. However, the functional role of auditory cortex in the retrieval of fear memories is unclear and how fear learning regulates cortical sensory representations is not well understood. To address these questions, we use acute optogenetic silencing and chronic two-photon calcium imaging in mouse auditory cortex during fear learning. Longitudinal imaging of neuronal ensemble activity reveals that discriminative fear learning modulates cortical sensory representations via the suppression of cortical habituation.

## Introduction

While the amygdala plays a critical role in associative fear conditioning, neural circuits in sensory cortex are also thought to contribute to the acquisition and expression of learned fear (LeDoux, 2000; Maren and Quirk, 2004; Herry and Johansen, 2014). For example, during associative auditory fear learning, lesions or pharmacological inactivation of auditory cortex have been reported to perturb the acquisition and retrieval of fear memories (Romanski and LeDoux, 1992; Boatman and Kim, 2006; Letzkus et al., 2011; Yang et al., 2016). However, there is some debate over whether cortical involvement is related to the complexity of auditory stimuli (i.e., simple tones vs. complex sounds) or the nature of the conditioning protocol itself (simple conditioning using single sounds vs. discriminative learning with two sounds) (Grosso et al., 2015).

Electrophysiological studies have reported that neurons in auditory cortex show enhanced responses to conditioned tones immediately after fear conditioning (Weinberger and Diamond, 1987; Quirk et al., 1997; Weinberger, 2004, 2015), leading to the proposal that changes in cortical sensory representations contribute to memory strength or stimulus discrimination. However, these findings are typically based on the responses of small subsets of recorded neurons that appear to show learning-related changes in activity. Furthermore, the inability to record from identified populations of cortical neurons over long time periods has made it difficult to determine how sensory representations are modified to support retrieval of fear memories.

Here, we use optogenetic activation of local GABAergic interneurons to rapidly and reversibly silence the auditory cortex (Olsen et al., 2012; Kato et al., 2015) in mice undergoing auditory fear conditioning. We show that auditory cortex plays a critical role in discriminative fear learning. We also take advantage of chronic two-photon calcium imaging in primary auditory cortex (A1) of awake mice (Kato et al., 2015, 2017) to examine cortical sensory representations before and after discriminative fear learning. We find that CS- representations are selectively reduced following fear conditioning, while CS+ representations are maintained. We show that this decline in CS- responses can be accounted for by habituation, a non-associative form of learning that weakens responses to repeatedly experienced stimuli which lack behavioral relevance (Kato et al., 2015). Taken together, these results indicate that discriminative fear learning regulates cortical sensory representations by preventing habituation to conditioned stimuli.

## Materials and Methods

### Animals and Surgery

All procedures were in accordance with protocols approved by the UCSD Institutional Animal Care and Use Committee and guidelines of the National Institutes of Health. Mice were acquired from Jackson Laboratories (*GAD2-Cre* [JAX 010802], *PV-Cre* [JAX 008069], *SOM-IRES-Cre* [JAX 013044], *VGAT-ChR2-EYFP* [JAX 014548], *ROSA-LSL-tdTomato* [JAX 007908], and *Ai32-ChR2* [JAX 012569]) and housed in a room with a reversed light cycle. Experiments were performed during the dark period.

Adult mice (5–16 weeks old, male and female) were anesthetized with isoflurane and injected with dexamethasone (i.p., 2 mg/kg). A stainless steel headbar was fixed to the skull with glue and dental acrylic. The muscle overlying the right auditory cortex was removed, and a 2 × 3 mm craniotomy was made leaving the dura intact. Virus was injected at 10–16 locations (centered at 2.3 mm from bregma, 3.8 mm from midline), 120–250 μm from the pial surface, at 30 nl/site. For imaging L2/3 pyramidal cells, we injected AAV 2/9-syn-GCaMP6s (1.9 × 10^12^ GC/ml) in mice heterozygous for *GAD2- Cre* and *Rosa-LSL-tdTomato*. For imaging PV and SOM cells, we injected AAV 2/9-syn-FLEX-GCaMP6s (2.9 × 10^12^ and 5.5 × 10^12^ GC/ml, respectively) in mice heterozygous for *PV-Cre* or *SOM-Cre* and *Rosa-LSL-tdTomato*. All viruses were from the University of Pennsylvania Vector Core. A glass window (a coverglass with a ∼300 μm-thick glass plug) was placed over the craniotomy and the edges were sealed with 1.5% agarose. The window was secured with dental acrylic. Baytril (10 mg/kg) and buprenorphine (0.1 mg/kg) were injected before mice were returned to their home cages. Mice were imaged 3–7 weeks following virus injection.

### Intrinsic Imaging

The primary auditory cortex was mapped with intrinsic signal imaging as described previously (Kato et al., 2015, 2017). Briefly, intrinsic signal images were captured with a tandem lens macroscope and 12 bit, CCD camera (CCD-1300QF, VDS Vosskühler) from isoflurane- and chlorprothixene- anesthetized mice. Images of surface vasculature were acquired using green LED illumination (530 nm) and intrinsic signals were recorded (27 Hz) using red illumination (615 nm). Each trial consisted of 1-s baseline followed by a 1-s sound stimulus (75 dB pure tone with a frequency of 3, 10, or 30 kHz, 10–20 trials for each frequency) and 30-s inter-trial interval. Images were averaged across trials and Gaussian filtered. The vasculature patterns of the intrinsic signal map were matched to those under the two-photon microscope to ensure imaging was in A1.

### Mouse Behavior

Following headbar implantation, mice were water deprived to no more than 80% of their free-drinking weight. In addition, mice were handled daily and habituated to head-fixation in a sound-attenuating enclosure over a period of 5–14 days. Once habituated to head-fixation, mice were placed in front of a lick port with IR detector and trained to lick freely for water for periods of >30 min. During discriminative fear conditioning experiments, animals were presented with two 30-s, frequency-modulated tones (5 Hz modulation rate), 1 octave apart. Tones (70 dB) were presented from a calibrated, free-field speaker (ES-1, TDT) positioned approximately 5 cm from the left ear. An inter-trial interval (ITI) of 30–60 s (5 s jitter) separated tone presentations. During fear conditioning, mice were given a 1–2 s tail shock (0.2–0.5 mA) that co-terminated with the presentation of one of the two tones (CS+). The fear learning protocol spanned 4 days: Day 1, Preconditioning with four trials of each tone, Days 2 and 3, Conditioning with ten trials of each tone (total of 20 CS+/US pairings), Day 4, Retrieval (Post-conditioning) with four trials of each tone. Imaging experiments followed the identical protocol. For optogenetic experiments, two of the four CS+ and CS- trials on Day 4 were combined with cortical LED illumination. Simple fear conditioning with a single tone used the same 4 day protocol (Day 1, four trials of tone alone; Days 2 and 3, ten conditioning trials each day; Day 4, two of the four trials were combined with LED illumination). Lick rate was calculated as licks per second for the CS+, CS-, and ITI. Because the ITI varied in length, ITI lick rate was determined from the 15 s period at the center of the ITI for each trial. Tones and lick signals were generated using behavior software (BControl^1^) running on a real-time Linux system.

For optogenetic experiments, VGAT-ChR2 or PV-ChR2 mice were implanted with a glass window overlying the right auditory cortex as used for imaging. A calibrated electrostatic speaker (EC1, TDT) coupled to a custom-made stainless steel earphone was directed to the left ear canal. For unilateral cortical inactivation, light pulses (10 ms, 20 Hz) were delivered via a fiber-coupled LED (∼20 mW, 470 nm, 1 mm fiber, 0.48 N.A.; Doric lenses) positioned 1–2 mm above the cranial window.

### Two-Photon Calcium Imaging

Awake mice were head-fixed under a two-photon microscope, GCaMP6s and tdTomato were excited at 920 nm (Mai Tai, Newport), and images (512 pixels × 512 pixels covering ∼500 μm × 500 μm) were acquired with a commercial microscope (B-scope, Thorlabs) running Scanimage software using a 16× objective (Nikon) at 28.4 Hz. Images were acquired from L2/3 (120–250 μm below the surface).

Mice were conditioned while head-fixed under the two-photon microscope using the same protocols described above for behavioral experiments. To reduce potential movement-related changes in cortical activity (Schneider et al., 2014; Zhou et al., 2014), we imaged mice pre- and post-conditioning without a lick port (*n* = 27). We confirmed that imaging did not interfere with discriminative fear learning (*n* = 2 mice). In a subset of L2/3 pyramidal cell experiments (*n* = 4), AM tones separated by 0.5 octaves were used. Experiments using these conditions were not obviously different and results were pooled. Subsequently, PV and SOM cell experiments used AM tones separated by 0.5 octaves.

### Image Analysis

Lateral motion during imaging experiments was corrected by cross-correlation alignment and regions of interest (ROIs) for visually identifiable cells were manually drawn. Neuropil contamination was corrected by subtracting the fluorescence signal from a ring-shaped background ROI drawn around the cell body from the fluorescence signal of the cell body as previously described (Kato et al., 2015, 2017).

Cells were judged as significantly excited if they fulfilled two criteria that take into account the variable timecourse and trial-to-trial reliability of sound-evoked activity. First, dF/F traces had to exceed a threshold of excitation for a minimum of 170 consecutive frames during the tone presentation (6-s, 20% of tone) on 75% of trials. Since the half decay time of dF/F responses to single action potentials is 1–2 s (Chen et al., 2013), this 6-s time window minimizes the possibility that spontaneous activity will be classified as an evoked response. The threshold for excitation was calculated for each cell as 1 × standard deviation of baseline activity in the 10-s preceding the tone. This threshold was determined using an ROC analysis to yield a 90% true positive rate (Kato et al., 2015). Second, the average trace from all trials had to exceed the excitation threshold for a minimum of 170 consecutive frames (6 s). Because inhibitory responses tend to be smaller than excitatory responses, the threshold for inhibition was set to 0.5 × standard deviation of baseline activity. Imaging fields in which ≤2% of cells responded to the CS+ or CS- tones were excluded.

The area above baseline of the dF/F signal was used in the calculation of the change index for neurons across conditioning. Change index was defined as (area Post - area Pre)/(area Pre + area Post). Only cells that were judged as responsive were included in this analysis.

Pyramidal cells were classified as discriminating if they met the following criteria. First, the smoothed traces for the CS+ and CS- responses during tones had to be significantly different as determined using the Wilcoxon signed-rank test on ≥75% of trials. Second, the difference in the area under the curve for the trial-averaged traces of the CS+ and CS- had to exceed a threshold of 0.2. This threshold was chosen empirically to prevent misclassification of differences between traces due to any fast transients (<1 s) remaining after smoothing.

The Euclidean distance between the population vectors of the CS+ and CS- was calculated using the following formula:

Euclidean distance = (q1−p1)2+(q2−p2)2+....+(qn−pn)22

where *q* equals a cell’s averaged response to the CS+ and *p* equals a cell’s averaged response to the CS-. Only cells that were classified as discriminating were included in this analysis.

## Results

**FIGURE 1:**
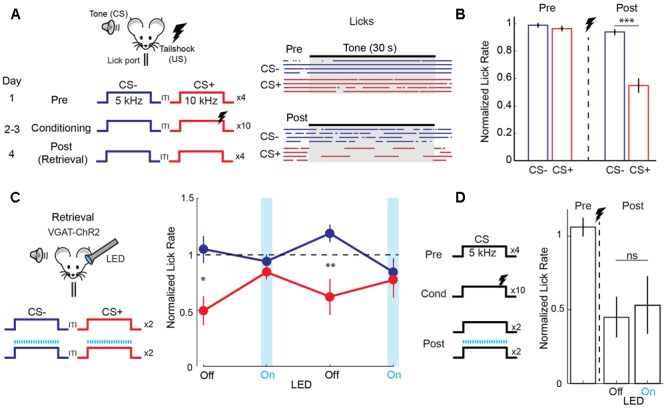
Auditory cortex is required for discriminative fear learning. **(A)** (Left) Schematic of auditory fear conditioning protocol. ITI, inter-trial interval; US, unconditioned stimulus. (Right) After conditioning, there is a selective reduction in licking during the CS+. Raster plot shows individual licks during CS– (blue) and CS+ (red) trials for one mouse on the day before (Pre) and after (Post) conditioning. **(B)** Summary data (*n* = 32 mice) showing selective reduction in lick rate (normalized to ITI rate) during CS+ tones on the post conditioning day (paired *t*-test, *p* < 0.001). **(C)** Cortical inactivation blocks expression of discriminative fear learning. (Left) Schematic for cortical silencing during memory retrieval in VGAT-ChR2 mice. Blue tics, LED flashes. (Right) Trial by trial analysis of lick rates (normalized to ITI lick rate) for control (LED off) and cortical inactivation (LED on) trials (*n* = 9 mice). Mice respond differently to CS+ and CS– tones on control trials (paired *t*-test, *p* = 0.035, 1st LED Off trial; *p* = 0.008, 2nd LED Off trial), but not on inactivation trials (*p* = 0.349, 1st LED On trial; *p* = 0.544, 2nd LED On trial). **(D)** Cortical inactivation has no effect on memory retrieval during simple (non-discriminative) fear learning using only one tone. (Left) Simple fear conditioning protocol. (Right) Summary data of lick rates to CS tones before (Pre) and after (Post) conditioning. Lick rates are reduced similarly whether the LED is off or on during interleaved CS trials (*n* = 5 mice, paired *t*-test, *p* = 0.637). Error bars are SEM. ^∗^*P* < 0.05, ^∗∗^*P* < 0.01, ^∗∗∗^*P* < 0.001, ns, not significant.

We studied discriminative fear learning in head-fixed mice trained to obtain water at a lick port (**Figure 1A**). One of two FM tones (30 s duration, one octave difference) was paired with a mild tail-shock (CS+) while the other was left unpaired (CS-) and learning was measured as the fear-induced suppression of licking (**Figure 1A**). The day after conditioning, mice suppressed licking during the CS+ but not the CS- tone (**Figure 1B**). We tested the role of auditory cortex in fear memory retrieval using optogenetic silencing in mice expressing the light-activated cation channel channelrhodopsin-2 (ChR2) in cortical GABAergic interneurons (Olsen et al., 2012; Kato et al., 2015). Unlike lesions or pharmacological manipulations, this approach offers precise temporal control of cortical activity in individual animals on a trial-by-trial basis. Following normal conditioning, unilateral cortical LED illumination was applied during interleaved presentations of the two tones on the day of memory retrieval (**Figure 1C**). Under these conditions, acute cortical silencing abolished fear behavior during CS+ tones and responses during CS+ and CS- tones were identical (**Figure 1C**). Since cortical inactivation in the absence of tone presentation had no effect on lick rate (*n* = 4 mice, paired *t*-test, *p* = 0.512), these results indicate that auditory cortex is necessary for memory retrieval after discriminative fear learning. One potential explanation is that cortical inactivation blocks the ability to detect tones. To address this possibility, we tested the effect of cortical silencing in mice that underwent simple fear conditioning with a single tone (**Figure 1D**). However, during memory retrieval in these mice, fear triggered by the conditioned tone was unaffected by cortical inactivation. Taken together, these results using acute and reversible silencing indicate that auditory cortex is necessary for tone discrimination, but not for tone detection or fear memory retrieval itself.

**FIGURE 2:**
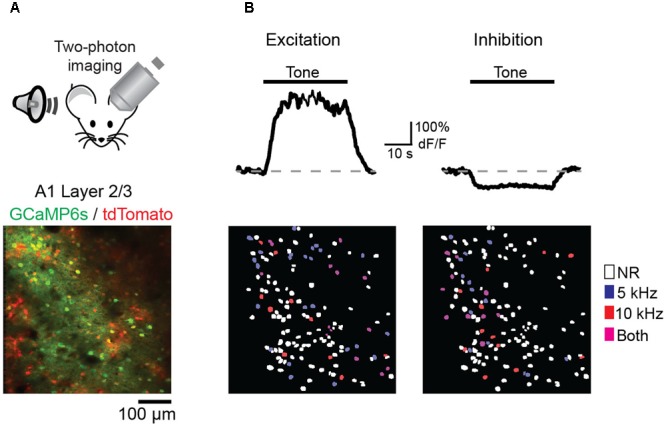
Imaging A1 sensory representations in awake mice. **(A)** (Top) Recording schematic. (Bottom) *In vivo* image of GCaMP6s in layer 2/3 pyramidal cells (green) and tdTomato-expressing interneurons (red). **(B)** (Top) Example traces of GCaMP6s responses in two pyramidal cells showing tone-evoked excitation (left) and inhibition (right). (Bottom, left) Spatial map of cells in one imaging field with significant excitatory responses to either the CS– (blue), CS+ (red), or both tones (purple). Non-responsive cells (NR) marked as white. (Right) Same as left panel, but for cells with significant inhibitory responses.

**FIGURE 3:**
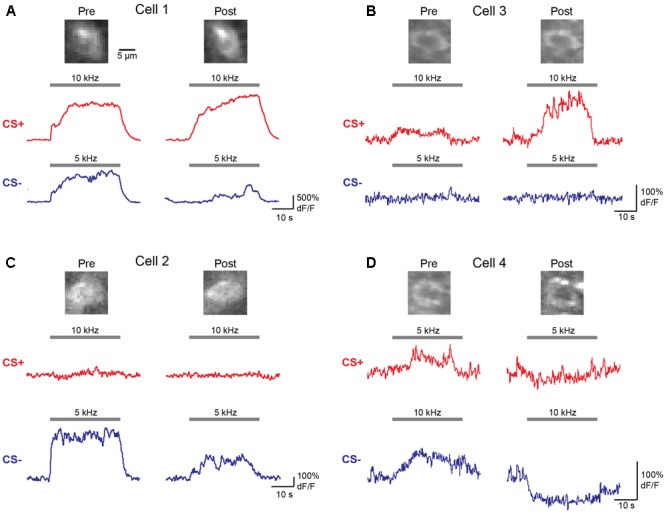
Responses of individual L2/3 pyramidal cells before and after fear conditioning. **(A–D)** Averaged dF/F responses to CS+ (red) and CS– (blue) tones before (Pre) and after (Post) conditioning are shown for four individual cells from two mice. Top, images of the same cells on Day 1 (Pre) and Day 4 (Post) of the identical fear conditioning protocol used for behavioral analysis. Traces show responses to the tones indicated by gray bars.

**FIGURE 4:**
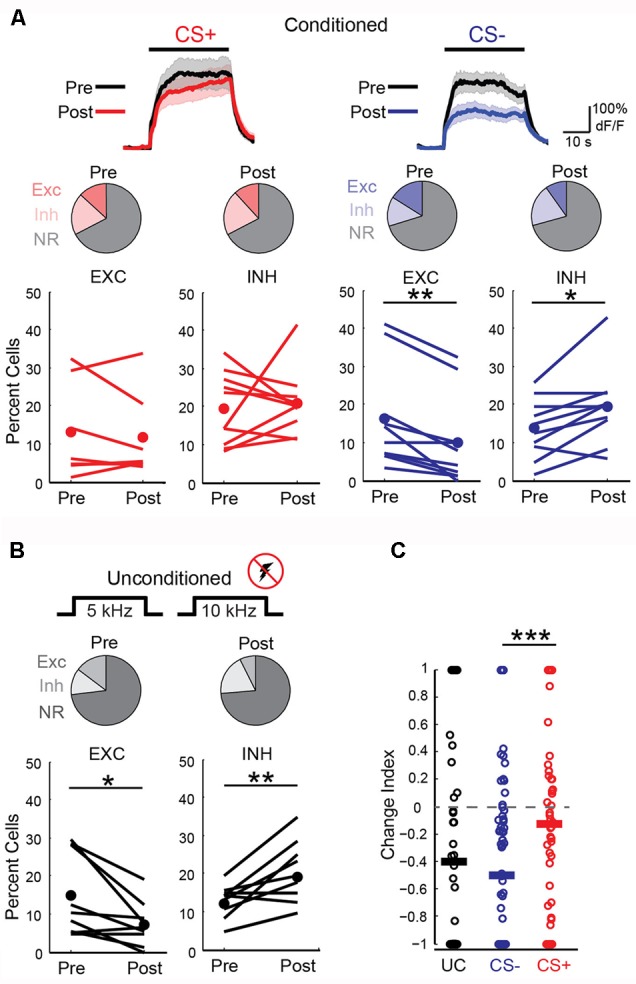
Fear conditioning prevents habituation of cortical sensory representations. **(A)** Left, responses to CS+ tones are similar before and after fear conditioning. Top, average CS+ tone response (*n* = 66 cells, 10 mice) before (Pre) and after (Post) conditioning. Line, average; shading, SEM. Middle, pie charts show fraction of cells with significant excitation (Exc), inhibition (Inh), or no response (NR) before (13, 20, and 67%, respectively) and after (12, 21, and 67%) conditioning. Bottom, fear conditioning does not change fraction of cells excited (EXC) or inhibited (INH) in individual mice (Paired *t*-test, excitation: *p* = 0.550, *n* = 7 mice; inhibition: *p* = 0.718, *n* = 10 mice). Lines, individual mice; filled circles, average. (Right) Excitation to CS– tones is significantly reduced after conditioning. Top, average CS– response of all cells (*n* = 88 cells, 10 mice). Middle, fraction of Exc, Inh, and NR CS– cells before (16, 14, and 70%, respectively) and after (10, 20, and 71%) conditioning. Bottom, fraction of cells excited by CS– tones decreases while fraction that are inhibited increases (Paired *t*-test, excitation: *p* = 0.001; inhibition: *p* = 0.014). **(B)** In a cohort of unconditioned mice (*n* = 9) that experienced the same protocol without shock, tone-evoked excitation decreases and inhibition is enhanced. Pie charts show fraction of cells with significant excitation (Exc), inhibition (Inh), or no response (NR) on Day 1 (13, 12, and 75%, respectively) and Day 4 (7, 19, and 74%). Bottom, fraction of cells excited or inhibited in individual mice (Paired *t*-test, excitation: *p* = 0.035; inhibition: *p* = 0.006). **(C)** Change index reveals a reduction in the magnitude of excitatory responses to CS– (*n* = 88 cells) but not CS+ tones (*n* = 66 cells, *n* = 10 mice, two-sample *t*-test, *p* < 0.001) following fear conditioning. Unconditioned mice experiencing the same tones show a reduction in response strength (*n* = 9 mice, 77 cells, two-tailed *t*-test, *p* < 0.001) virtually identical to CS– responses in conditioned animals (two-sample *t*-test, *p* = 0.385). Bars, average. ^∗^*P* < 0.05, ^∗∗^*P* < 0.01, ^∗∗∗^*P* < 0.001.

We next wished to determine the impact of discriminative fear learning on representations of conditioned tones in auditory cortex. We used adeno-associated virus (AAV) vectors to express the calcium indicator GCaMP6s (Chen et al., 2013) in auditory cortex neurons of transgenic mice containing the activity independent reporter tdTomato in GABAergic neurons (*GAD2-IRES-Cre* × *Rosa-LSL-tdTomato*). This allowed us to optically distinguish glutamatergic pyramidal cells (green) from GABAergic interneurons (green + red) (**Figure 2A**). Two to three weeks following virus injection and the implantation of a glass window over auditory cortex, we performed intrinsic signal imaging to map the precise position of A1 (Kato et al., 2015). Using this approach, we imaged responses to the CS+ and CS- tones before and after fear conditioning in layer 2/3 (L2/3) pyramidal cells of awake mice (**Figure 2A**). Prior to conditioning, the two tones elicited an enhancement or suppression of pyramidal cell activity (**Figure 2B**, 331/815 cells, *n* = 10 mice), consistent with previous reports of sustained tone-evoked excitation and inhibition in auditory cortex of awake animals (Wang et al., 2005; Sadagopan and Wang, 2010; Kato et al., 2015). Furthermore, a significant subset of cells (29%) responded to both tones. We tracked the responses of individual cells to CS+ and CS- tones before and after discriminative fear conditioning (**Figure 3**). In contrast to reports of enhanced tone responses following simple fear conditioning (Quirk et al., 1997; Weinberger, 2004, 2015), discriminative learning under our conditions caused no change in the average fraction of pyramidal cells responsive to the CS+ tone (**Figure 4A**). Rather, the fraction of cells excited by the CS- tone decreased and there was an increase in suppressed cells. Furthermore, conditioning also strongly reduced the average magnitude of excitation evoked by the CS- tone while there was no net change in the CS+ response (**Figure 4C**). At face value, these results suggest that discriminative fear conditioning selectively weakens cortical representations of CS- tones. However, in another cohort of mice undergoing the same protocol but without tone-shock pairing, we observed the same reduction in tone responses (**Figures 4B,C**). This weakening in responsiveness is identical to the selective habituation of responses to repeated, passively experienced auditory stimuli recently reported in A1 (Kato et al., 2015). Thus, the simplest explanation for our results is that, rather than enhancing CS+ responses, fear learning prevents the habituation of cortical representations for these salient tones.

**FIGURE 5:**
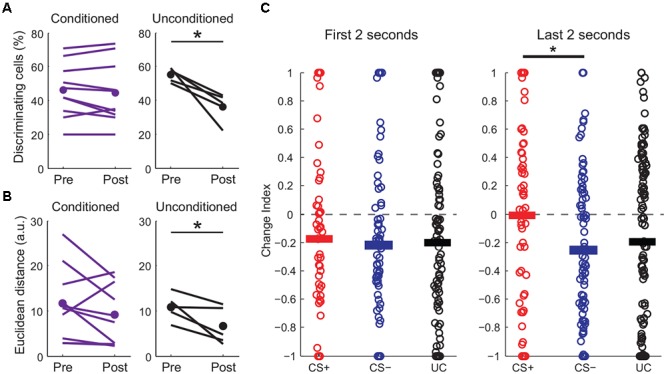
Fear conditioning modulates CS+ and CS– sensory representations. **(A)** Fraction of discriminating cells decreases in unconditioned (paired *t*-test, *p* = 0.016) but not in conditioned mice (two-sample *t*-test, *p* = 0.47). **(B)** Euclidean distance between tone population vectors is reduced in unconditioned animals (paired *t*-test, *p* = 0.048) but is maintained in conditioned animals (paired *t*-test, *p* = 0.176). ^∗^*p* < 0.05, ^∗∗^*p* < 0.01, ^∗∗∗^*p* < 0.001. **(C)** Change index showing the strength of excitatory responses during the first (left) or last (right) 2 s of tone presentation for conditioned (CS+ and CS–) and unconditioned (UC) mice. Although overall CS+ and CS– responses are reduced similarly at the beginning of the tone, CS+ responses are significantly stronger than CS– responses during the last 2 s of the tones (paired *t*-test, *p* = 0.017, *n* = 10 mice).

Perceptual acuity can be modulated by fear learning and this process is thought to involve cortical circuits (Li et al., 2008; Aizenberg and Geffen, 2013). We next made additional measures of how fear learning modulates cortical sensory representations. First, we examined pyramidal cells with distinct responses to CS+ and CS- tones and found that the fraction of these “discriminating cells” remained constant before and after fear conditioning (**Figure 5A**). In contrast, discriminating cells were markedly reduced in unconditioned mice that passively experienced the same tones. We further measured differences in population activity by calculating the Euclidean distance between population vectors of responses to CS+ and CS- tones. Although this cannot address variability and correlations in activity on individual trials, it does allow comparison of the state of the population for different stimuli. We found that while the Euclidean distance between tone population vectors were similar before and after fear conditioning, there was a significant reduction in distance between the population vectors for tones in unconditioned animals (**Figure 5B**). This measure suggests that while sensory representations remained distinct with fear conditioning, they became less separable when the same tones were passively experienced. Interestingly, similar to previous findings using unit recording (Quirk et al., 1997), the difference in excitatory responses to the CS+ and CS- after conditioning was larger toward the end of the tones (**Figure 5C** and traces in **Figure 4A**) suggesting an anticipatory influence on cortical activity.

**FIGURE 6:**
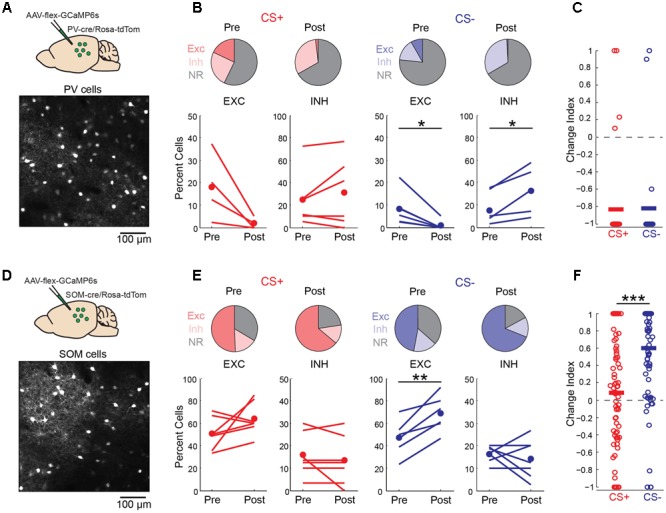
Discriminative fear conditioning selectively enhances SOM cell responses to CS– tones. **(A)** Top, GCaMP6s targeting approach in PV-cre mice. Bottom, *in vivo* image of GCaMP6s-expressing PV cells. **(B)** A decrease in PV cell tone-evoked excitation to both CS+ and CS– tones after fear conditioning. Top, fraction of PV cells with excitatory (EXC), inhibitory (INH) or no response (NR) to CS+ and CS– tones before (Pre) and after (Post) conditioning. CS+ Pre (18, 25, and 57%, respectively) and CS+ Post (2, 31, and 67%); CS– Pre (8, 18, and 74%) and CS– Post (<1, 33, and 67%). Bottom, fraction of cells shown separately for individual mice in response to CS+ (paired *t*-test, excitation: *p* = 0.088, *n* = 4 mice; inhibition: *p* = 0.300, *n* = 6) and CS– (paired *t*-test, excitation: *p* = 0.021, *n* = 6; inhibition: *p* = 0.029, *n* = 5). **(C)** Change index reveals a reduction in PV cell response strength to CS+ (*n* = 6 mice, 38 cells) and CS– (*n* = 6 mice, *n* = 24 cells; two-sample *t*-test, *p* = 0.927). Bars, average. **(D)** Top, GCaMP6s targeting in SOM-cre mice. Bottom, *in vivo* image of SOM cells. **(E)** SOM cell responses to CS+ tones are unchanged while excitation to CS– tones is enhanced following conditioning. Top, fraction of SOM cells with excitatory (EXC), inhibitory (INH) or no response (NR) to CS+ and CS– tones before (Pre) and after (Post) conditioning. CS+ Pre (51, 16, and 34%) and CS+ Post (64, 13, and 23%); CS– Pre (47, 16, and 37%) and CS– Post (69, 14, and 17%). Bottom, fraction of cells shown separately for individual mice in response to CS+ (paired *t*-test, excitation: *p* = 0.145; inhibition: *p* = 0.331, *n* = 7 mice) and CS– (paired *t*-test, excitation: *p* = 0.004; inhibition: *p* = 0.531). **(F)** Change index reveals an increase in SOM cell response strength to CS– but not CS+ tones (*n* = 7 mice, 78 cells; two-sample *t*-test, *p* < 0.001). ^∗^*p* < 0.05, ^∗∗^*p* < 0.01, ^∗∗∗^*p* < 0.001.

Local GABAergic interneurons in auditory cortex are thought to play an important role in both fear learning (Letzkus et al., 2011; Sarro et al., 2015) and habituation (Kato et al., 2015). Consistent with this idea, we observed a marked increase in neurons that were inhibited by the CS- tone following fear conditioning (**Figure 4A**). We thus considered the possibility that learning-related changes in interneuron activity could underlie the effects of fear conditioning on pyramidal cell activity. To address this question, we imaged the two main classes of cortical GABAergic neurons (Tremblay et al., 2016), parvalbumin (PV)- and somatostatin (SOM)-expressing interneurons, using conditional expression of GCaMP6s in PV-cre and SOM-cre mice (**Figures 6A,D**). For PV cells, fear conditioning led to a decrease in the fraction of cells excited by the CS+ and CS- tones as well as a marked decrease in response magnitude for both tones (**Figures 6B,C**). This similar reduction in activity in response to CS+ and CS- tones makes it unlikely that learning-related changes in PV cell activity accounts for the differences in pyramidal cell CS+ and CS- representations following fear conditioning. In contrast, conditioning led to distinct changes in SOM cell responses to CS+ and CS- tones. In particular, SOM cell excitation to CS- tones was markedly enhanced while responses to CS+ tones showed little net change (**Figures 6E,F**). At face value, these results suggest that the selective enhancement of SOM cell activity may account for the reduction of pyramidal cell responses to CS- but not CS+ tones following conditioning. However, we do not exclude the possibility that PV cells or other interneuron subtypes contribute to changes in cortical activity during fear conditioning in some fashion.

## Discussion

We found that the auditory cortex is necessary for discrimination of CS+ and CS- tones during memory retrieval and that fear conditioning regulates the discriminability of cortical sensory representations. Discriminative fear learning does not increase cortical representations of the CS+, *per se*, but rather sustains CS+ representations while CS- responses are reduced. The reduction in CS- responses mirrors the habituation of cortical representations to passively experienced tones lacking behavioral salience (Kato et al., 2015). Recent studies indicate that inhibitory circuits contribute to the induction of auditory fear learning (Letzkus et al., 2011; Lovett-Barron et al., 2014). Similarly, we show that learning related changes in SOM cell activity may help to regulate CS+ and CS- sensory representations during fear memory retrieval.

The amygdala receives sensory input from both the auditory thalamus and auditory cortex and lesion studies have led to the idea that simple fear conditioning can be mediated by either pathway (Romanski and LeDoux, 1992; LeDoux, 2000). It has been further proposed that the involvement of auditory cortex is dependent on the complexity of auditory stimuli (LeDoux, 2000). Consistent with this idea, pharmacological cortical inactivation during conditioning with FM sweeps has been found to reduce discriminative fear learning (Letzkus et al., 2011). However, more recent pharmacological studies using simple fear conditioning with single tones also found that cortical inactivation blocked both the acquisition and expression of learned fear (Yang et al., 2016; Banerjee et al., 2017). We used acute and reversible optogenetic silencing to specifically test the role of auditory cortex in fear memory retrieval. We found that optogenetic cortical inactivation did not block fear expression when learning was based on a simple fear conditioning protocol. In contrast, cortical silencing disrupted fear memory retrieval during discriminative fear learning. Since we used unilateral silencing, it is conceivable that the cortex ipsilateral to sound delivery was capable of processing information related to the conditioned tones. Nonetheless, under our conditions of unilateral cortical silencing, the auditory cortex appears to play an essential role in the discrimination of CS+ and CS- sounds.

Longitudinal imaging of A1 activity revealed that fear conditioning maintained the discriminability of cortical sensory representations to CS+ and CS- tones. In contrast, cortical representations became less distinct in animals exposed to the same tones without conditioning. This is likely due to the fact that across the population of L2/3 pyramidal cells, tone responses in unconditioned animals and CS- responses were diminished while CS+ sensory representations were sustained. There are a number of factors that could account for differences between our results and those of previous studies reporting increases in CS+ responses (reviewed in Weinberger, 2004, 2015). First, we studied the responses of the same identified L2/3 pyramidal neurons over days in awake mice while previous electrophysiological studies monitored unidentified cell types before and after conditioning across multiple cortical layers. We cannot exclude the possibility that fear conditioning selectively enhances responses to CS+ tones in deeper cortical layers. Furthermore, electrophysiological studies typically describe cortical neurons that show only transient responses at tone onset (i.e., Zhou et al., 2014). In contrast, we studied cortical activity using the same prolonged tones (30 s) typically used in rodent fear conditioning protocols. We specifically studied cells with sustained activity since cells with sustained responses must be those relevant for conveying information regarding the prolonged CS+ tone. While our calcium imaging approach could underestimate small differences in spike firing during tone onset, it should provide a good readout of sustained changes in firing activity underlying prolonged tones.

In A1, passive sound experience causes habituation of L2/3 sensory representations that reflects a selective upregulation of SOM cell activity (Kato et al., 2015). Furthermore, sound-guided behavior reverses these effects, suggesting that sensory representations are bidirectionally modified based on the behavioral relevance of sensory stimuli. Here we show that discriminative fear conditioning leads to a selective increase in SOM cell activity to CS- but not CS+ tones. The most parsimonious explanation for our results is that CS- sensory representations experience habituation while CS+ sensory representations are maintained. Ultimately, our results indicate that discriminative fear conditioning regulates cortical sensory processing by preventing habituation. This interplay of associative and non-associative learning processes ensures that cortical representations of salient sensory stimuli are retained while representations of stimuli lacking relevance can be reduced.

## Author Contributions

SG performed *in vivo* imaging and behavior experiments, analyzed data, and wrote the manuscript. HK assisted in data analysis. MJ and ML assisted in behavioral experiments and analysis. JI supervised the project.

## Conflict of Interest Statement

The authors declare that the research was conducted in the absence of any commercial or financial relationships that could be construed as a potential conflict of interest.

## Footnotes

1 www.brodylab.org
